# Fluorescence intensities of composite resins on photo images

**DOI:** 10.1007/s10266-020-00583-z

**Published:** 2021-02-03

**Authors:** Ioannis Brokos, Minos Stavridakis, Panos Lagouvardos, Ivo Krejci

**Affiliations:** 1 Division of Cardiology and Endodontology, University Clinics of Dental Medicine (CUMD), University of Geneva, 1, rue Michel-Servet, 1211 Geneva 4, Switzerland; 2 Athens, Greece; 3 Department of Operative Dentistry, School of Dentistry, National and Kapodistrian University of Athens, Athens, Greece

**Keywords:** Composite resin fluorescence, UVA light fluorescence, Photographic images, Blue light fluorescence, Direct fluorescence visualization

## Abstract

Recording fluorescence using flash photography, may help reduce time of capture and apply effectively in clinical practice. To test methods for visualizing composite resins fluorescence by direct digital photography. Sixty-four specimen discs (1.5 × 10 mm) were prepared from 8 different composite resin brands. Their CIELAB color coordinates (L*, a*, b*) and fluorescence were measured using a portable colorimeter and a fluorescence spectrophotometer. The mean of three measurements was recorded and then specimens were photographed by a DSLR camera with two different filters (365 nm and 405 nm) mounted on a commercial macro flash. RGB values of all specimens on the photographs were measured by using Photoshop software and converted to CIELab. Data were then analyzed using Kruskal–Wallis and Dunn’s multiple comparisons tests. Correlation and regression analyses were also used to relate fluorescence and color parameters on the photographs at *α* = 0.05. Fluorescence and color data indicated significant differences among the materials (*p* < 0.05). L* a* and b* color coordinates from both photographs were highly correlated to fluorescence intensities found by the reference method (*r*_365_ − 0.95, *r*_405_ − 0.94), while regression analysis indicated a strong linear relationship (*R*^2^_365_ − 0.88, *R*^2^_405_ − 0.89). The study showed that filtered flash photography either by the use of a 365 nm or a 405 nm band pass filter can directly visualize fluorescence of composite resin materials and differences in fluorescence between them.

## Clinical implication

The presented and documented method to disclose fluorescence is straight forward, cost effective and may be used in every routine dental office. As proved by this study, it may reliably disclose fluorescence of restorative materials and allows also for comparison between different materials’ brands. As reported in the literature, fluorescence documentation is not only useful for restorative materials. It may be used as well to identify dental plaque and calculus accumulation on teeth or to record demineralized lesions on tooth surfaces.

## Introduction

Color matching between natural dentition and composite restorative materials is critical for esthetic restorations [1–3]. Hue, chroma, and value or L*, a* and b* in color systems are the principal parameters that influence the esthetic outcome of a tooth-colored restoration [4, 5]. Contemporary enamel and dentin direct composite materials mimic the color gamut of enamel [6] and dentin [7]. However, materials of different brands, even of the same shade, vary not only in color [8] but also in fundamental optical properties that influence the overall appearance of the restoration, such as translucency [9], opalescence [10], illuminant metamerism [11], and fluorescence [12].

Many researchers agree on the importance of fluorescence as an optical property that could determine the esthetic success or failure of the restorative treatment [13]. Fluorescence is defined as the optical property of a substance that, while exposed to the exciting irradiation, absorbs the light and consequently emits this light at a longer wavelength [14–16]. Natural teeth show fluorescence, with dentin being 3 times more fluorescent than enamel mainly due to its organic collagen components [17]. The phenomenon of fluorescence in human dentition occurs under natural or artificial lighting (black light, night club settings, and galleries). Fluorescence of dental structures increases brightness and enhances the visual impression of vitality as part of a living tissue. Aesthetic restorative materials should present fluorescence properties similar to the tooth in order to mimic natural structures and therefore any variation in emission levels is undesirable for the restorations in the aesthetic zone [18–20]. Unfortunately, fluorescence intensities of dental tissues and restorative materials are not visually recognized and thus not controlled in clinical routine.

Studies on fluorescence in dentistry are aiming at two important issues. The first is related to fluorescence intensity levels of restorative materials introduced in the market and the second with the method of documenting this fluorescence not only in research labs but in clinical practice as well. A number of studies have addressed fluorescence intensity of commercially available composites and found significant differences among them and between them and tooth structures [21–23]. Tabatabaei et al. [24] showed that the thickness, type and brand of composite systems had a strong influence on their fluorescence properties. Park et al. [25] confirmed their results since they found that color changes of composite resins under F2 (4200^0^ k) or A (2800^0^ k) illuminations were probably due to their fluorescence and the interaction of illuminant light on these materials. Yu and Lee [26] showed perceptible color and fluorescence differences between flowable and composite resin materials of different brands. Kim et al. [27] compared the fluorescence intensities of various composites with the intensity of adjacent teeth and found that fluorescence differences were greater than on images taken under normal daylight. However, there are studies [28, 29] which demonstrated that most composites had comparable fluorescence intensities to that of human dentition which diminished after accelerated aging.

In the above studies, fluorescence was measured in a research lab using expensive and complex equipment like fluorοmeters, fluoro-spectrophotometers or UV–visible light reflectance spectrophotometers. No direct measurement of fluorescence of any kind in a routine clinical setting has been done so far. Since fluorescence is an optical phenomenon and considering that clinicians generally are familiar and relatively confident working with digital single lens reflex (DSLR) cameras for dental photography, some publications reported the documentation of fluorescence on digital photographs [30–41]. In some of them fluorescence images of composite resins were taken in a dark environment or in a black box, illuminated by a UV source, but not in an operating room under daylight illumination [30, 31, 33, 34, 41]. Gambonera and Blatz [30] were perhaps the first who recommended the use of fluorescence images within the dental operatory and during the restoration process. Instead of using standard continuous UV lights, Guzy and Clayton [35] recommended the use of UV-LED flashes on a photographic camera. Hein et al. [36] a customized flashlight at 365 nm, while Brokos et al. [40] developed a common xenon flashlight with an interchangeable 365 nm band pass filter. It is evident that several investigators have successfully used fluorescence phenomena within the dental operatory, but the chosen UV light was different and the equipment in many cases was customized without the necessary validation of the fluorescence intensities measured on the photographs. Hung and Tuan [42] showed that 365 nm, 380 nm and 405 nm are all suitable for exciting fluorescence emission of teeth. In some of the above studies no information was given on the peak wavelength of the excitation light used [32–34, 37], in some the peak was reported at 365nm [36, 40], in others at 380 nm-385nm [38, 41], at 395nm [35] and 405nm [39]. Although 365 nm light is the wavelength the least hazardous to human skin and eyes in the field of UVA radiation, the use of safer (longer wavelengths) that may excite teeth and dental materials for fluorescence emission is preferred but has not been tested for direct visualization of fluorescence.

The purpose of the study was to evaluate the ability of a commercial digital photographic camera to record correctly and reveal on photographs the fluorescence of some direct dental composite resins, by using filters with different excitation peaks mounted on a commercial macro photo flash. Color coordinates of the emission intensities recorded on the photos will be compared to reference fluorescence intensities. The zero hypothesis was that color coordinates on photographs were not related with fluorescence values taken with standard spectrophotometric methods.

## Materials and methods

For the purpose of the study 8 discs (10 mm ± 0.1 mm in diameter and 1.5 ± 0.1 mm thickness each) were prepared from 8 different direct dental composite resins. Detailed composition based on manufacturers' data are shown in Table 1. Sample size estimation was based on an effect size of 0.60, *α* = 0.05 and 1 − *β* = 0.90 using G-Power v3.1.7 (Franz Faul, Universität Kiel, Germany). Samples were prepared carefully to avoid air entrapment and polymerized with a large area tip of a LED light curing device (Mini Led Black, Satelec Acteon) at 1250 mW/cm^2^ for 20 s from the top and 20 s from the bottom surface. Specimens’ thickness was confirmed by a micrometric gauge and their surface was visually examined for any defect. Visible surface irregularities or differences in thickness higher or lower than 0.1 mm resulted in the exclusion of the specimen. The discs remained dry in a dark chamber and 48 h before the measurements they were put into distilled water bath at 37 °C. A portable colorimeter (FRU-WR18, Shenzhen Wave Optoelectronics Technology) with D65 (Noon daylight of 6504 K) light source, with a measuring window of 4 mm in diameter an observer angle of CIE 10° and a repeated accuracy of Δ*E* < 0.06, was used to measure the CIELAB coordinates (L*, a* and b*) on a black and a white background. Three measurements were taken of the same surface of each sample, at its central area and the average values were recorded as the mean primary color parameters of each sample.

**Table 1:** Detailed information of the materials used in the study

Material (code)	Company	Type	Composition	Filler Loading	Shade	Exp. date	Batch No
Essentia (A)	GC dental corp	mh	UDMA, Bis-MEPP, silicon dioxide, fluoro-alumino-silicate glass	76 wt% (63 vol%)	Dark enamel	2020-01	1601121
Herculite XRV ultra (B)	Kerr corp	nh	Bis-GMA, TEGDMA, barium glass filler (0.4 μm) silicon dioxide (0.02–0.05 μm)	78 wt%	A3	2022-02	7090524
Opallis (C)	FGM Produtos Odontologicos	mh	BisGMA, BisEMA, TEGDMA, (resin matrix = 21–22.5%) and barium glass, aluminum silicate and silica dioxide	77.5–79 wt%	EA3	2021-02	270218
Herculite classic (D)	Kerr corp	mh	Inorganic fillers average particle size 0.6 μm	79 wt%	A3	2020-03	6323214
G-aenial (E)	GC dental corp	mh	UDMA & dimethacrylate co-monomers, strontium lanthanide fluoride pre-polymerized fillers, silica, fumed silica, 16–17 μm (400 nm strontium glass, 100 nm lanthanide fluoride, 16 nm silica)	76 wt% (62 vol%)	A3	2021-10-02	1810031
TPH spectrum (F)	Dentsply/caulk	mh	BisGMA, BisEMA, TEGDMA, Barium aluminum borosilicate glass (mean particle size < 1 μm) and highly dispersed silicon dioxide (particle size 10–20 nm)	77 wt% (57 vol%)	A3	2021-10-31	1.81E ± 09
Inspiro (G)	Edelweiss DR	nh	Barium alumino fluoride glass, (size 0.02–2 μm), Bis-GMA based	83 wt%	skin neutral	2021-10	201234
Mosaic (H)	Ultradent	nh	Zirconia–silica glass ceramic and nanometer silica fillers	56 vol%	Α3	2020-09	BG777

*mh* microhybrid, *nh* nanohybrid

Color differences of specimen pairs under the different illumination conditions were calculated using Eq. 1.

1 $$\Delta E*_{ab} = [\left( {L*_{{1}} - L*_{{2}} } \right){2} \pm \left( {\left( {a*_{{1}} - a*_{{2}} } \right){2} \pm \left( {b*_{{1}} - b*_{{2}} } \right)} \right]^{{{1}/{2}}}$$

Specimens were also measured with the use of a reflectance spectrophotometer (Ci64 UV, X-Rite Inc) to record the highest fluorescence intensity of the materials in the area between 400 and 700 nm with and without a 365 nm band pass UV filter.

**Fig. 1:**
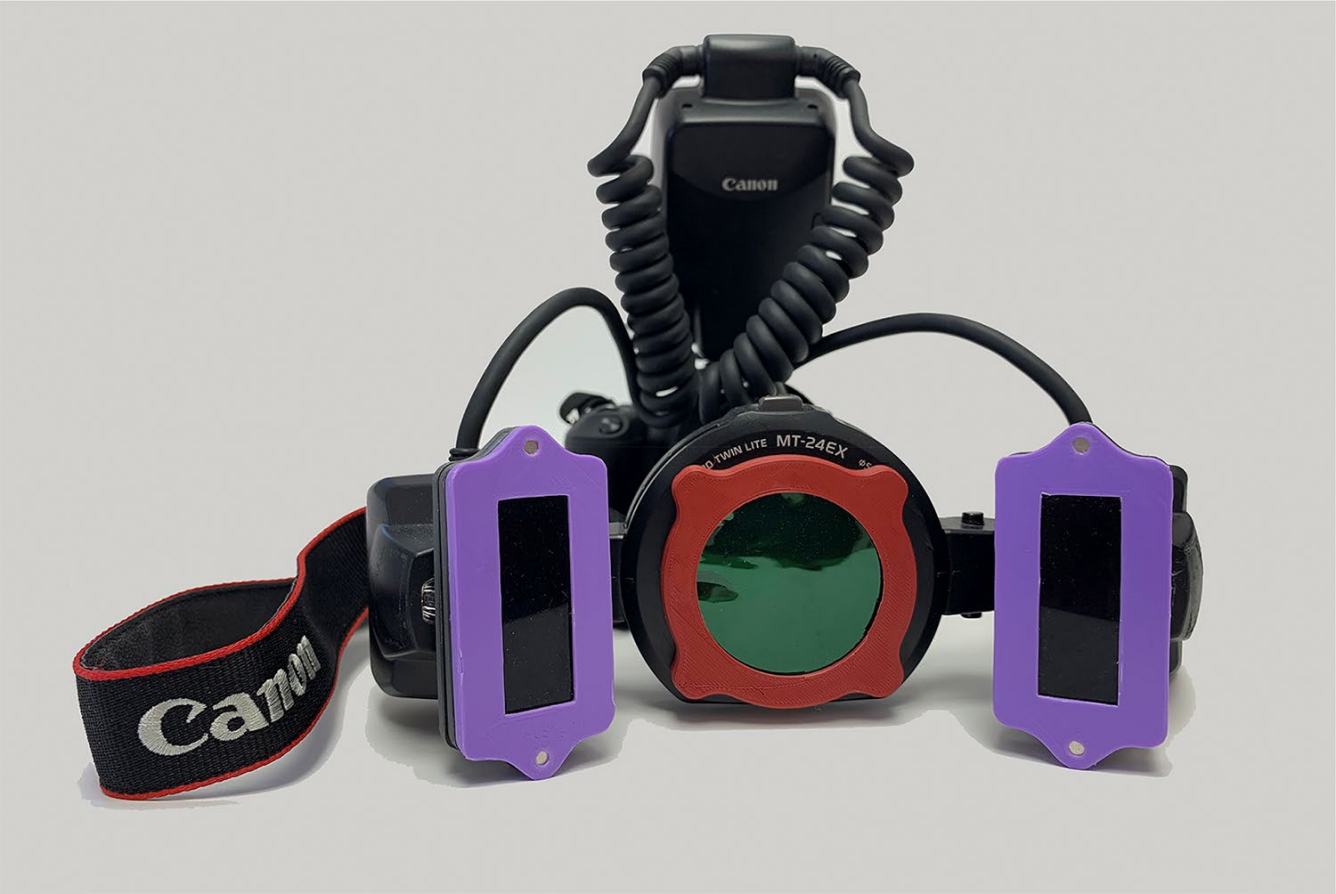
Camera set up used for the study. Violet colored plastic indicates the UV filters on flash lamps and red plastic the green filter on the lens

Finally, eight specimens, one from each composite resin, were photographed on the same frame using a DSLR camera (Canon EOS 200D, Canon, canon.com) with a twin flash (Canon MT-24EX Macro Twin Lite flash) on the sides of a macro lens (Canon EF, 100-mm f/2.8 Macro USM lens), at a magnification of 1:2.5 (Fig. 1). Two different photographs were taken. One with a 365 nm band pass glass filter (range from 250 to 400 nm) over both flashlights (UVA induced blue fluorescence) and a second with a 405 nm band pass glass filter (range from 395 to 410 nm) over the flashlights (Violet induced green fluorescence) along with a double layered circular green gelatin filter (540 nm max of a band width from 460 to 680 nm /RS4460, Rosco) in front of the camera lens. The first image was captured with f/14, ISO 800, 1/125, AWB and the second with f/10, ISO 1600, 1/125, AWB, both in jpeg format.

These RGB images were opened in Photoshop CS3, v.10 (Adobe, San Jose CA, USA) and by defining a round selection area of 62,591 pixels (10.0 × 10.0 units corresponding to 2.78 × 2.78 mm) in the center of each sample of the images, median R, G and B parameters of the area were obtained three times, always repositioning the selection area. Their average was recorded as the mean specimen’s value for each parameter. These RGB values were converted to XYZ and then to CIE L* a* b* values using the color converter (EasyRGB, IRO group Ltd, www.easyrgb.com).

Statistical tests (Kruskal–Wallis nonparametric ANOVA with post hoc multiple comparisons tests) were used to analyze the data for differences in fluorescence between brands at a level of significance *α* = 0.05. Pearson's correlation and Regression analyses were also calculated to define the type and strength of association of resin fluorescence with and L*, a*, and b* parameters, taken either by the colorimeter or measured on the photographs. For the analyses, MedCalc statistical package v.10.2.0 (MedCalc Software) was used.

## Results

Based on collected data, initial estimation of sample size and power was very conservative since the calculated effect sizes for fluorescence, colorimetric and photographic data were higher (6.898 for FL values, 1.536 for CLM values, 3.190 for P365 values and 5.137 for P405 values). This increases the power of all analyses to 0.999–1.000.

**Fig. 2:**
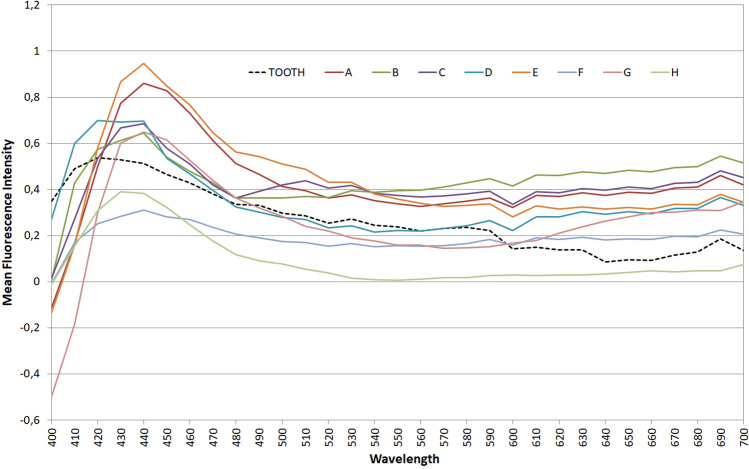
Mean fluorescence intensities of all brands (at 400–700 nm). Black dashed line indicates the intensity of a normal human tooth

Fluorescence data are given in Table 2. The highest intensities were found at 440 nm for all materials (Fig. 2). Kolmogorov–Smirnov test revealed normality of data (*p* > 0.05) while Bartlett's test indicated equal variances (*p* > 0.05). One-way ANOVA indicated significant differences among the different brands (*p* < 0.0001) which located by Bonferroni multiple comparisons test and shown in Table 2. CIE L*, a* and b* color coordinates of the materials under CIE-D65 standard illuminant, were calculated. In Table 2 only the values of L* parameter are shown with the results of the analysis for differences among materials (Kruskal–Wallis non parametric ANOVA and Dunn’s post hoc multiple comparisons test at *α* = 0.05) which indicated significant differences among the materials.

**Table 2:** Mean ± SD fluorescence intensities of composite resins and their CIE L* values measured by a colorimeter (under D65 illumination) or on their photos either with 365 nm (P365) or 405 nm band-pass filter (P405) on camera flash (*n* = 8)

Material	Fluorescence Intensity	L* values Colorimeter	L* values P365	L* values P405
A	0.98 ± 0.21_a_	61.23 ± 0.16_bc_	83.85 ± 0.87_a_	50.50 ± 1.42_ab_
B	0.64 ± 0.14_bcd_	62.27 ± 0.19_bc_	65.62 ± 0.99_cde_	31.29 ± 2.95_bc_
C	0.69 ± 0.25_abc_	63.17 ± 0.25_ab_	72.82 ± 3.10_abcd_	42.84 ± 1.50_ab_
D	0.78 ± 0.21_abc_	63.06 ± 0.15_abc_	78.38 ± 1.15_ab_	50.39 ± 1.19_a_
E	0.95 ± 0.15_ab_	64.12 ± 0.41_ab_	79.69 ± 5.34_abc_	51.38 ± 1.28_a_
F	0.35 ± 0.06_d_	66.99 ± 0.22_ab_	54.92 ± 1.07_e_	10.49 ± 0.88_c_
G	0.74 ± 0.30_abc_	63.14 ± 0.75_abc_	66.91 ± 1.66_bcde_	44.44 ± 2.98_abc_
H	0.44 ± 0.17_ cd_	61.15 ± 0.34_c_	59.19 ± 1.01_de_	14.36 ± 0.95_c_

Same letters in cells of the same column indicate no significant differences at *α* = 0.05

**Fig. 3:**
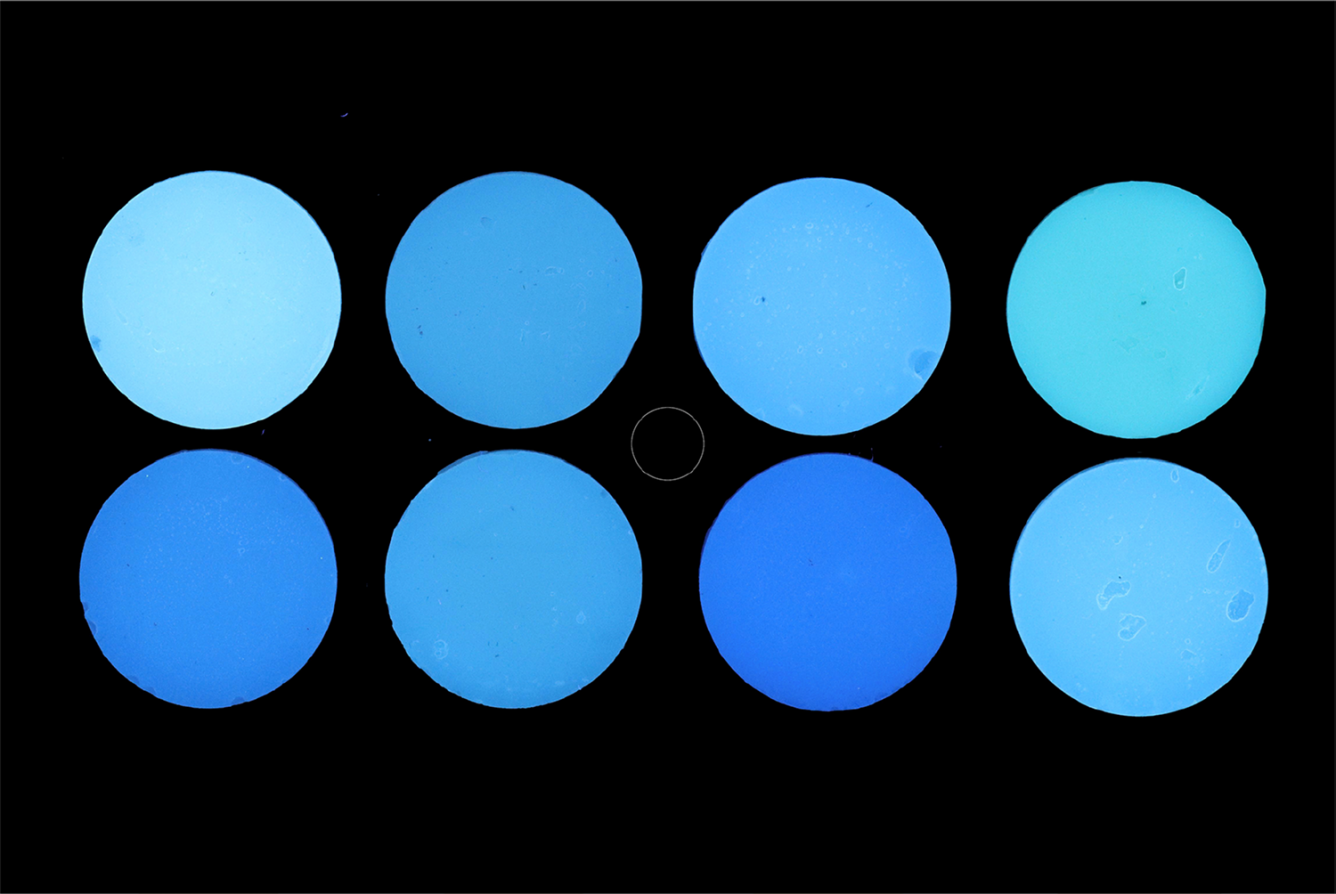
Photo images of 8 different composite resin samples taken with the use of a 365 nm filter on the flash. Upper row: A,B,C,D. Lower row: H,G,F,E. White circle in the middle is the standard selection area for the measurements

**Fig. 4:**
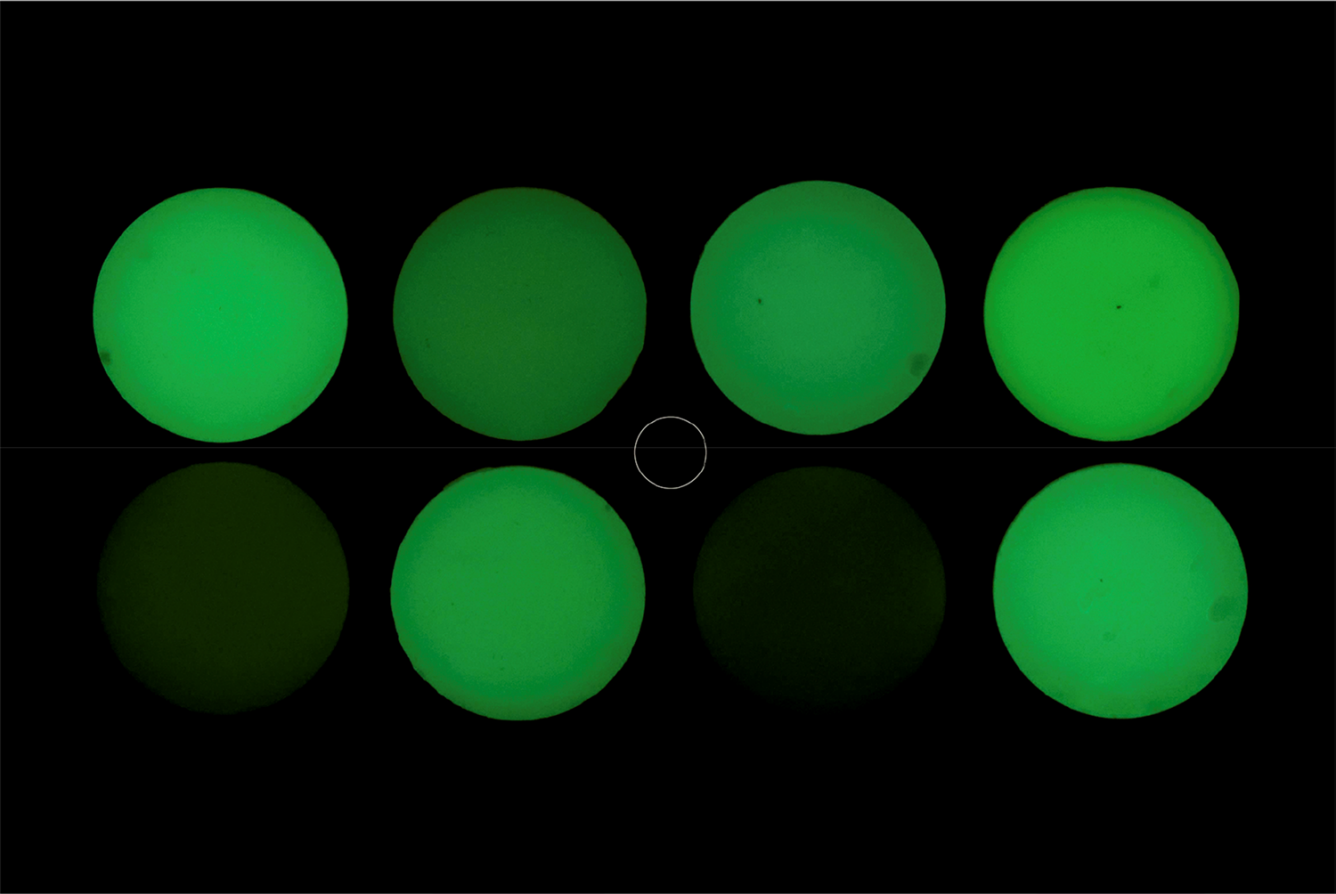
Photo images of 8 different composite resin samples taken with the use of a 405 nm filter on the flash and a green filter on the lens. Upper row: A,B,C,D. Lower row: H,G,F,E. White circle in the middle is the standard selection area for the measurements

CIE L* a* b* values, converted from RGB values, from photographs with 365 nm and 405 nm filter, respectively, on the flash, were also collected. In Table 2, the values of L* parameter measured on the photographs are also shown with the results of the statistical analysis for differences between materials based on Kruskal–Wallis non parametric ANOVA and Dunn’s post hoc multiple comparisons test at *α* = 0.05. The significant difference between the materials found by the analysis indicates a good discrimination power for differences in brightness of composite resins on photographs. Representative RGB images of the samples, taken by both methods, are shown in Figs. 3 and 4.

**Fig. 5:**
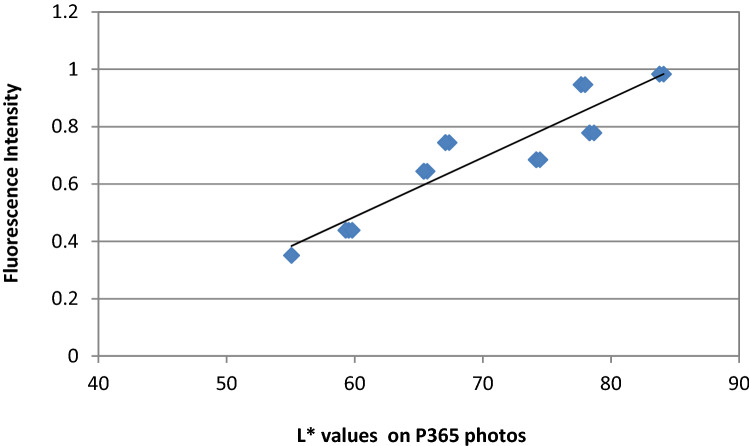
Regression line of fluorescence intensity and L* values on P365 photos. Regression’s coefficient of determination is high (*R*^2^ = 0,876) with a linear equation *y* = 0.0207*x* − 0.7537

**Fig. 6:**
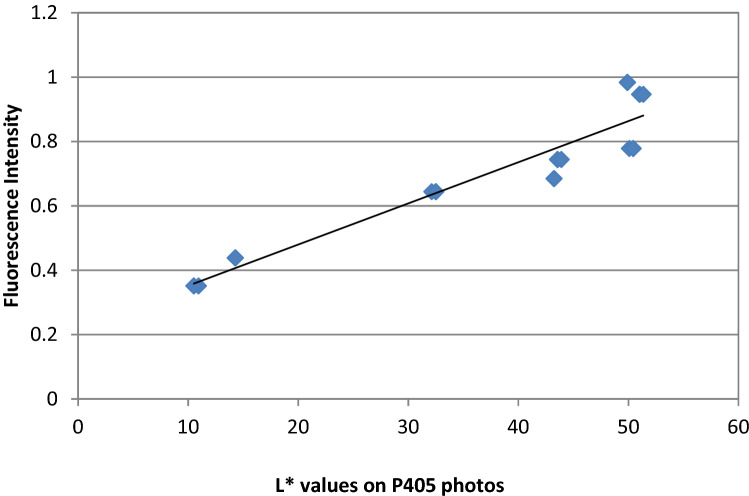
Regression line of fluorescence intensity and L* values on P405 photos. Regression’s coefficient of determination is high (*R*^2^ = 0,888) with a linear equation *y* = 0.0128*x* − 0.2239

Results from correlation analysis for the association of color data between the two differently filtered images and the colorimetric or fluorescence data for all color coordinates are shown in Table 3. Figures 5 and 6 present the regression lines of the highly correlated L* parameters in photographs with fluorescence intensities.

**Table 3:** Correlation coefficients (*r*) between measurement methods, for L* a* and b* color coordinates

Pairs	L*	a*	b*
P365-P405	0.929753	0.932977	0.959552
P365-CM	−0.37762	−0.4315	0.013596
P365-Fluor	0.952468	−0.84831	0.917908
P405-CM	−0.30179	−0.34617	−0.13088
P405-Fluor	0.945154	−0.93438	0.867282
CM-Fluor	−0.37604	0.304019	0.060423

P365 means photos taken with a 365 nm filter over the flash light. P405 photos with a 405 nm filter. CM means colorimeter, Fluor means Fluorescence values taken by the fluorescence spectrophotometer

## Discussion

The study rejected the hypothesis of no association of color coordinates values on photos with fluorescence values taken by standard spectrophotometric methods. Both filtered flash techniques gave photos with highly correlated values of their color coordinates with fluorescence intensities measured by a UV–Vis spectrophotometer.

Fluorescence data of composite resin systems recorded by the spectrophotometer showed variations in their intensities. According to this study, composites A and E showed the highest fluorescence values, composites B, C, D and G showed intermediate fluorescence similar to the tooth, while materials F and H had the lowest fluorescence intensities. Variations in fluorescence intensities of composite resins were also shown in previous studies [10, 22, 23, 26, 28, 29, 34, 43]. The highest peak for all restorative materials appeared at 440 nm, while for the tooth the peak was at 420 nm. The highest fluorescence intensity for restorative materials at 440 nm was also found in the study of Tavares et al. [43] who used the same excitation light filtered at 365 nm, and in studies of Takahashi et al. [28] and Jablonski et al. [29] who, however, used an excitation light at 380 nm. We must mention that in all the above studies, there was no attempt to relate the differences in fluorescence to compositional differences of the materials. Since no information is given by the manufacturers concerning what substance they are using to imitate natural tooth fluorescence, more studies are needed to relate composition and structure of composite materials to their fluorescence properties.

Studies on dental composite resins have used different exciting lights to record their fluorescence. Tavares et al. [43] used 365 nm and recorded fluorescence emission at 440–450 nm, Takahashi et al. [28] and Jablonski et al. [29] used 380 nm for excitation and observed emission at 440 nm, Meller and Klein [22], da Silva et al. [34] and Meller and Klein [23] used 398 nm for excitation and recorded emissions at 452 nm and 485 nm. Excitation/emission wavelengths for composite resins are guided by the fluorescence behavior of dental tissues (enamel and dentin), since composite resins should mimic the optical properties of tooth structure. Normal tooth structure emits fluorescence at 430–450 nm or 480–500 nm when excited by light at 337 nm, at 460 or 560 nm when excited by light at 375 nm and at 520–540 nm when excited by light at 405 nm [19, 44, 45]. Differences between methods are due to differences in the devices, methodology and the materials used for the recording of fluorescence. It is known that only a part of the exciting radiation is emitted as fluorescence and in order to produce the maximum fluorescence, the exciting light has to be at its maximum excitation wavelength. Otherwise an enhancement of fluorescence intensity or its signal is needed to make the phenomenon more intense. The lower the wavelength, and the narrower the spectrum of the exciting light around its maximum, the stronger the fluorescence intensity will be. Laser beams, xenon lights and LEDs are used as excitation light sources. White LEDs and xenon lights have a wide spectrum and must be filtered. In our study we have used xenon-filtered light used in everyday dental documentation photography, since it is available in most dental offices. The filter that was used in the first series of photographs was a 240–400 nm band pass filter and the resulted images presented strong blue lighted composite surfaces on a dark background with visible differences in brightness. Analysis showed an almost perfect correlation of L* values in photographs with the fluorescence values measured by the spectrophotometer, under a strong linear relationship. This means that visualization of fluorescence was successful by applying this method and that fluorescence values can be predicted from the photographic values of L* according to the regression equation *y* = 0.020*x* − 0.753.

Other attempts to record fluorescence by a photographic camera have also been reported [21, 27, 33, 34, 36, 37, 39–41, 46, 47]. Although they all succeeded to present photographs of teeth and restorative materials lighted by UVA irradiation, in none of these a reference method was used and for this reason we have no indications on the relationship of lightness in the photographs to material fluorescence. In two of the studies [33, 34] the digital images were analyzed by a computer software program (ScanWhite DMC/Darwin systems, Brazil) with limited information on how the program related RGB/Lab differences to fluorescence. Our study showed also a high correlation of UV–Vis fluorescence values to a* or b* values on the photographs, which, however, cannot be used for the visualization of fluorescence with the existing set-up. A red or a yellow filter in front of the lens may be helpful in visualizing fluorescence in these areas of the spectrum, and therefore, further studies are needed on this subject.

The band pass filter we have used in the first series of photographs permitted only to UVA and half of the UVB spectrum, contained in xenon light source, to reach the materials for excitation. Since exciting lights of higher wavelengths are considered safe, a second series of photos were taken with a band pass filter at 405 ± 5 nm. This wavelength of light excites for fluorescence normal tooth structures in the region of 520–540 nm (green area of the visual spectrum), in lower intensities than blue fluorescence, but with a higher sensitivity for enamel fluorescence and an increased difference of the relative fluorescence between tooth structure and composite resins [20, 27]. To record only the fluorescence in the green area, a green band pass filter of 540 nm (460–680 nm) was used in front of the lens. The resulted images presented green-lighted composite resin surfaces of an intermediate tone on a dark background, but with visual differences in brightness levels between the materials. Data analysis indicated a very strong correlation of L* values in the photos with fluorescence data taken by the UV–Vis spectrophotometer (*r* = 0.95) and with the L* data on the blue images (*r* = 0.94). This relation was found to follow a strong linear curve with the equation *y* = 0.012*x* + 0.223, meaning that fluorescence is highly predicted by the L* values on these photos, as on 365 nm photos.

The methods for visualization of fluorescence of dental materials presented in this study may arise some questions concerning the safety of handling and using UV light in front of patient or personnel. Two answers can be given. One is that the use of a filtered flash (365 nm) which allow UVA and UVB light, already contained in the source, to pass in the excited light without enhancing its intensity, cannot be considered more harmful than the unfiltered flash, used in everyday dental photography. The second is that the duration of a flashlight is extremely short (only 1/40,000 s) and practically is considered safe. For these reasons the use of a common xenon or LED white flash light is probably safer than UV continuous light sources, or specially designed UV flash lights. Since lights of 405 nm are even safer than most UV radiation [45], the use of flashes filtered with 405 nm is practically with no effect on human tissues. However, wearing UV protection eyeglasses for patients, clinicians and personnel is advised as an appropriate precaution. This precaution should be followed not only when the filtered 365 nm flash is used but also with 405 nm or any other strong white light.

The methods for visualization of fluorescence of dental materials presented in this study may arise some questions concerning the safety of handling and using UV light in front of patient’s face. Two answers can be given. One is that the use of a filtered flash (365 nm) which allows UVA and UVB rays to pass freely, cannot be considered more harmful than the unfiltered flash used in everyday dental photography, since they are already contained in it. The second is that the duration of a flashlight is extremely short (only 1/40,000 s) and for this reason could be considered safe. The use of a common xenon or LED white flashlight are probably safer than UV continuous light sources, or specially designed UV flash lights. Lights of 405 nm are even safer than most UV radiation, and their use on flashes are no different than unfiltered ones. However, wearing UV protection eyeglasses for patients, clinicians and personnel is advised as an appropriate precaution for eye protection. This precaution should be followed not only when the filtered 365 nm flash is used but also with 405 nm or any other strong white light.

One of the strengths of the study is that it gives the equations of the relation of Lightness on the photos and fluorescence intensity of the materials, very useful in predicting fluorescence of materials from their lightness on photos (*y* = 0.020*x* − 0.753 for 365 nm filtered photos and. *y* = 0.012*x* + 0.223 for 405 nm filtered photos). However, both methods need to be validated in a greater scale of materials, shades and thicknesses. The study may have the limitation of using as reference intensities those found by a specific fluorescence measuring instrument. Although such instruments may be reliable, possible differences between them, may alter the regression equation found in our study, useful for predicting fluorescence accurately on photos. For this reason studies with different reference methods and even different brands of composite materials may be helpful. Another limitation is probably the thickness of the sample we have chosen. Although we have chosen the thickness of 1.5 mm which was reported to have a higher fluorescence intensity from 0.5 or 1.0 mm [43], more studies are needed for thicknesses 2.0 mm, 2.5 mm or 3.0 mm, which are also very common in clinical restorations.

The use of filtered flashes has the advantage of using the same commercial photographic equipment for many different applications without needing heavy and expensive extra equipment. Different filters allow information either on enamel or dentin structures, on plaque or calculus and of course on the presence of bacteria activity or products in areas we are interested in. Such filters are already implemented in several intraoral cameras, however, filtered flashes can also serve successfully such purposes, without any additional money or sophisticated equipment.

## Conclusions

Our current study has documented ultraviolet and violet induced direct fluorescence images of composite resins using a commercial digital photographic camera coupled with appropriate 365 nm or 405 nm band-pass filters over the flashes and a green filter on the lens. The L* values obtained on the photographs by the two methods were highly correlated and linearly related to fluorescence intensities recorded by a fluorescence spectrophotometer. Our findings allow us to conclude that the 405 nm filtered images are equally capable of showing fluorescence as the 365 nm filtered flash images and therefore can be used alternatively if there would be any doubt on the safety of using UV lights in clinical practice for direct visualization of fluorescence.
